# Motion Artifact Suppression for Insulated EMG to Control Myoelectric Prostheses

**DOI:** 10.3390/s20041031

**Published:** 2020-02-14

**Authors:** Theresa Roland

**Affiliations:** Institute of Biomedical Mechatronics, Johannes Kepler University, 4040 Linz, Austria; theresa.roland@jku.at

**Keywords:** motion artifacts, insulated/capacitive EMG, artificial intelligence, neural network, time domain features, myoelectric prosthesis

## Abstract

Myoelectric prostheses help amputees to regain independence and a higher quality of life. These prostheses are controlled by electromyography, which measures an electrical signal at the skin surface during muscle contractions. In this contribution, the electromyography is measured with innovative flexible insulated sensors, which separate the skin and the sensor area by a dielectric layer. Electromyography sensors, and biosignal sensors in general, are striving for higher robustness against motion artifacts, which are a major obstacle in real-world environment. The motion artifact suppression algorithms presented in this article, prevent the activation of the prosthesis drive during artifacts, thereby achieving a substantial performance boost. These algorithms classify the signal into muscle contractions and artifacts. Therefore, new time domain features, such as Mean Crossing Rate are introduced and well-established time domain features (e.g., Zero-Crossing Rate, Slope Sign Change) are modified and implemented. Various artificial intelligence models, which require low calculation resources for an application in a wearable device, were investigated. These models are neural networks, recurrent neural networks, decision trees and logistic regressions. Although these models are designed for a low-power real-time embedded system, high accuracies in discriminating artifacts to contractions of up to 99.9% are achieved. The models were implemented and trained for fast response leading to a high performance in real-world environment. For highest accuracies, recurrent neural networks are suggested and for minimum runtime (0.99–1.15 μs), decision trees are preferred.

## 1. Introduction

Electromyography (EMG) sensors are applied, beyond others, for myoelectric prosthesis control, diagnostic purposes and exoskeletons. The state-of-the-art dry sensors, which require a conductive connection to the skin, are typically applied for myoelectric prosthesis control. This research group has already published the developed flexible insulated EMG sensors [1,2] to overcome the disadvantages associated with dry conductive EMG sensors. The insulated sensors avoid pressure marks and are independent of sweat and the skin condition in general.

Various research has presented the control of high-level dexterity prostheses [3,4,5]. Well-established EMG features have been implemented [6,7,8] and different signal processing algorithms have been developed [9,10,11]. Although these algorithms achieve high accuracies for distinguishing hand movements, they often lack robustness in real-world environment [12,13]. However, in terms of practical application, robustness is preferred over technologically complex and unreliable systems [14].

The main reason for the lacking robustness are the motion artifacts [15], which have various origins. They typically occur in low frequency range. However, these artifacts also appear in the frequency range of the contraction EMG, which limits the possibilities for filtering. To achieve high robustness, real-time motion artifact suppression algorithms were implemented on an ultra-low-power microcontroller (μC) in this contribution. These algorithms aimed at an activation of the prosthesis drive solely during an actual contraction. The aim of the algorithm was the distinction of the two classes: contraction and artifact.

An algorithm with a frequency domain feature has already been presented by Roland et al. [16]. Frequency domain features require a Fourier transform, which leads to a long time span between decisions. The presented algorithm in Roland et al. [16] has a weakness in detecting short contractions. At the beginning and the end of a contraction, the muscle tissue moves relatively to the skin surface, which causes a motion artifact. As short contractions mainly consist of the beginning and the end of a contraction, a motion artifact is overlaid to the contraction EMG signal. However, these short contractions are of high importance to enable co-contractions, which switch the prosthesis movement mode.

In this article, only time domain (TD) features were implemented and instead of the linear separator in Roland et al. [16] different artificial intelligence models, such as neural networks (NN), were investigated. The algorithms were designed to confidently detect the short contractions. Additionally, the algorithms aimed at a robust detection of strong and weak contractions to allow a proportional control of the myoelectric hand prosthesis.

These algorithms were developed with MATLAB^®^ aiming at high accuracy but low calculation resources. New features were designed, and different artificial intelligence models were trained, and the resulting models were implemented on an ultra-low-power μC. The algorithms were designed for the insulated EMG sensor, however, they can also be applied to increase the robustness of conductive EMG sensors or even of other biosignal sensors.

## 2. Methods

**Figure 1:**
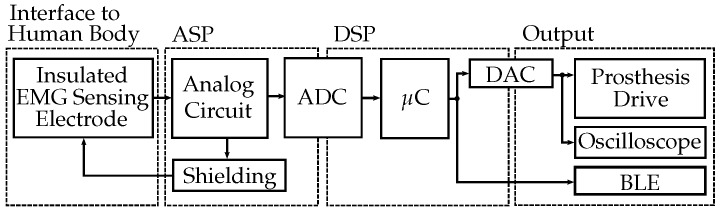
First, the EMG signal coupled from the human body is amplified and filtered by the analog circuit (ASP). In the μC, the signal is then digitally filtered and evaluated (DSP). The contraction EMG signal is provided at the prosthesis drive via the digital-to-analog converter (DAC). For experimental applications, the signal can be connected to the oscilloscope or Bluetooth (BLE) module (Adapted from Roland et al. [2]).

**Figure 2:**
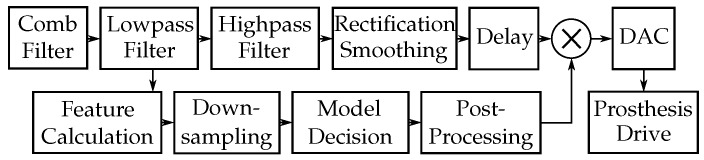
Signal flow of the digital signal processing. The decision of the model (0 for artifacts or 1 for contractions) is multiplied with the delayed processed EMG signal.

Figure 1 shows the signal flow graph of the capacitive EMG signal. The data acquisition, filtering, rectification and smoothing were described in detail by Roland et al. [1,2]. The block diagram of the digital signal processing is plotted in Figure 2. Additional data pre-processing, the feature calculation, the decision algorithm (logistic regressions, decision trees, NN or recurrent neural networks (RNN)) and the data post-processing as well as the signal delay are described in this section.

### 2.1. Data Acquisition and Pre-Processing

**Figure 3:**
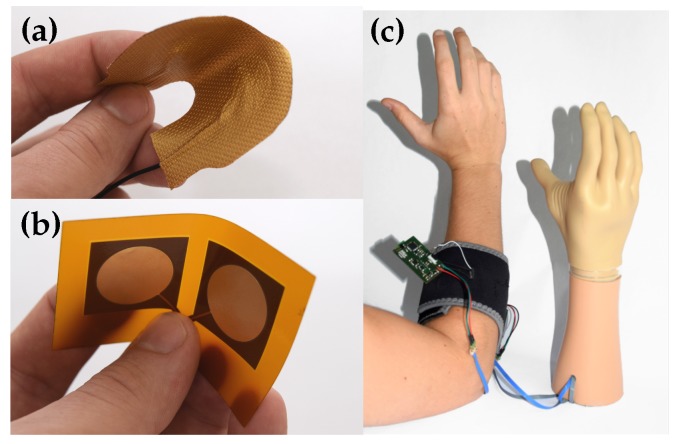
(**a**) Textile capacitive EMG sensing electrode. (**b**) Flexprint capacitive EMG sensing electrode. (**c**) Control of myoelectric hand prosthesis with capacitive EMG measurement setup (Adapted from Roland et al. [1]).

Insulated EMG data were acquired with the sensor developed by Roland et al. [1,2]. Figure 3 shows flexible coupling electrodes, which are an assembly of textiles or a flexprint. The EMG sensing system filtered the signal with a first-order analog bandpass ($f_{CL}$= 11 Hz, $f_{CU}$ = 1064 Hz), a digital comb (50 Hz and harmonics) and a first-order digital lowpass ($f_{C}$ = 531 Hz). In the feature calculation path, a second order digital high pass ($f_{C}$ = 60 Hz) was implemented. The optimal choice of these above-mentioned parameters was investigated in Roland et al. [1,2]. The parameter values were selected according to these findings. The flexible sensor was placed at the musculus extensor digitorum at the human forearm of one subject. Four different types of data were measured. Data for strong and weak isometric contractions, random sequences of short contractions and measurements of many artifacts were acquired. Therefore, various different motion artifacts were generated, such as sensor lift-off, external mechanical shocks or vibrations.

**Figure 4:**
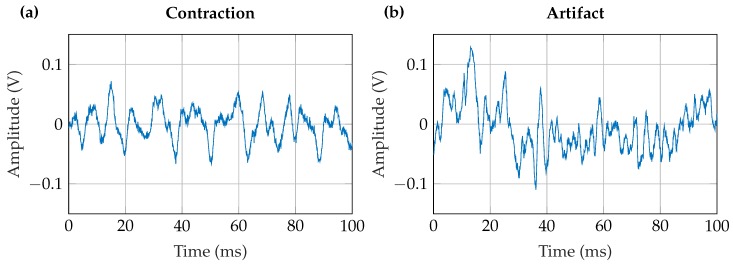
Raw signals measured with the capacitive EMG sensor at a sampling frequency of 10 kHz. (**a**) EMG signal of a strong contraction. (**b**) Exemplary artifact. Please note that artifacts are varying greatly, depending on its origin.

The data were measured with a digital oscilloscope (HS3 of TiePie Engineering) at the μC’s digital-to-analog converter. The 10 s measurements were sampled by the oscilloscope at 10 kHz to fulfill the Nyquist-Shannon sampling theorem [17]. A measured contraction and a motion artifact are presented in Figure 4. The measured EMG data were downsampled by averaging to 2 kHz. According to Roland et al. [2] downsampling to 2 kHz does not reduce the EMG signal quality. This sampling frequency of 2 kHz was applied for the feature calculation. Time windows with small signals, which would not lead to a prosthesis drive activation, were removed from the data set.

After these pre-processing steps, a total of 381 s of contractions and 489 s of artifacts were the remaining training, test and validation data. These data comprised a wide variety of different contractions and artifacts to achieve high robustness in real-world environment.

The contraction and artifact signals were mixed to 1 s long windows. The contraction and artifact signal windows were alternated, hence a fast reaction to the changing signal class was ensured. The features were calculated over these alternating time windows. Features which were slow at the transients led to errors at the transition from one class to the other. By iterating the classes in the data set, these slow features were consequentially removed by the feature selection. This is essential for real-world performance, where artifacts and contractions are alternating and a fast reaction of the decision is required. For the training and validation set of the RNN, the duration of the windows was varied for each snipped, thereby preventing the RNN to learn the periodic behavior of the training set.

The training (70%) and validation (15%) set amounted to 85% of the data. The validation data were selected out of the 85% of the data when training the models with cross validation, see Section 2.6. The remaining 15% were the test set, which was equal for all models. The training, validation and test set comprised an equal percentage of the strong, short, and weak contractions, and artifacts according to their share on the whole data set. A sample of the features highly correlates to the previous and next sample in time, the adjacent feature values are very similar. Drawing individual samples for the allocation to the training, validation and test data would lead to non-generalizable results. Therefore, continuous time sequences were selected in the allocation in order that the training, validation and test data are independent from each other.

### 2.2. Feature Calculation

In this contribution, new features and modifications of features for biosignal processing, and well-established features were included to the feature calculation. The TD features were hand-crafted as the models should achieve high accuracies with a shallow model architecture. The feature vector feat was calculated by filtering with a first-order lowpass, an exponentially decaying moving average filter (EMA) [18]:

(1) $$feat_{i}=\left(feat_{i-1}+f\left(x_{i}\right)\right)b\;,$$

where $f(x_{i})$ denotes a function of the current comb and lowpass filtered value $x_{i}$. The function $f(x_{i})$ is defined differently for each feature. The coefficient *b* was selected in the interval [0,1] (in floating-point format). This *b* was implemented as a fixed-point with a multiplication and a shift operation to avoid operations with floating-point variables. This implementation requires less calculation resources than a standard moving average filter [2].

The features were designed with the aim to detect differences of contraction EMG and artifacts. These TD features also attempt to detect frequency information, which is relevant for the distinction of the classes, e.g., by the zero-crossing rate. Some features were implemented several times with varied parameters, which enables the extraction of different signal characteristics.

The features were designed with MATLAB^®^ and implemented on the μC in C. The MATLAB^®^ and C implementation were in the same value range and truncation, overflow and saturation were considered. Hence, the values listed in the following section are valid for both implementations.

Some features were saturated at an upper bound (ub) and/or a lower bound (lb) to avoid a drift of the feature values, thus achieving a fast transition between the different classes. The selected parameters of the implemented features are listed in Table 1, Table 2, Table 3, Table 4, Table 5, Table 6 and Table 7.

#### 2.2.1. Zero-Crossing Rate (ZCR)

**Figure 5:**
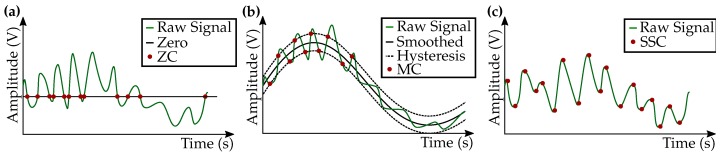
Exemplary sketches of features. Note that different variants of the sketched features were calculated. (**a**) Zero-Crossing (ZC): The crossings of the raw signal through the zero line are detected. (**b**) Mean crossing (MC): The crossings of the raw signal through the smoothed signal (+/− a hysteresis) are detected. (**c**) Slope Sign Change (SSC): The changes of the slope sign of the raw signal are detected.

The ZCR (Figure 5a) is a well-established feature for EMG signal analysis, which comprises frequency information. When the EMG signal crosses zero, the feature is increased by 100. This feature update parameter is set to 100 to prevent the feature variable from vanishing right away due to truncation caused by the shift operation in the lowpass filter in Equation (1). Moreover, the value was not selected higher to prevent overflows. The parameter was determined, so that no overflows occurred in the training data. These facts are also valid for the selection of this feature update parameter for the following described features. As also noise with low amplitude contributes to the feature, a hysteresis (hyst) was added to detect the crossings only, when the signal exceeds a certain amplitude. An upper bound ub was included in ZCR2S to avoid a drift of the feature value, thereby achieving fast transitions. The conditions for the ZCR were defined as

(2) $$f\left(x_{i}\right)=\left\{\begin{array}{ccc} 100, & [s=1]; & \mathrm{if}\;x_{i}>hyst\wedge s=0\\ 100, & [s=0]; & \mathrm{if}\;x_{i}<-hyst\wedge s=1\\ 0, & [s=s]; & \mathrm{else}\;, \end{array}\right.$$

where *s* denotes the sign of the previous zero-crossing.

#### 2.2.2. Mean Crossing Rate (MCR)

The contraction EMG is usually superimposed to a slight artifact at short contractions, due to the relative movement of the muscle tissue to the skin surface at the beginning and the end of a contraction. To detect this superimposed EMG signal, the crossing of the signal through the smoothed signal, as shown in Figure 5b, increases the feature by 100.

A hysteresis hyst was included to avoid noise contributing to the feature. A feature variant with a saturation at an upper bound (ub) and a lower bound (lb) was implemented. The conditions for the MCR were defined as

(3) $$f\left(x_{i}\right)=\left\{\begin{array}{ccc} 100, & [s=1]; & \mathrm{if}\;x_{D}>\left(v+hyst\right)\wedge s=0\\ 100, & [s=0]; & \mathrm{if}\;x_{D}<\left(v-hyst\right)\wedge s=1\\ 0, & [s=s]; & \mathrm{else}\;, \end{array}\right.$$

where *s* denotes the sign of the previous mean crossing and *v* is defined as

(4) $$v=(smo_{i})e\;.$$

The smoothed signal $smo_{i}$ is calculated with an EMA with the coefficient *c*:

(5) $$smo_{i}=\left(smo_{i-1}+x_{i}\right)c\;.$$

The input $x_{i}$ is delayed and scaled by

(6) $$x_{D}=\left(x_{i-delay}\right)d\;.$$

#### 2.2.3. Slope Sign Change (SSC)

When the EMG signal slope is changing (Figure 5c), the feature is increased by 100. To avoid noise contribution, the feature is calculated with the smoothed signal from Equation (5). Variants of the feature require the signal slope to maintain the direction for a minimum number of data points $d_{min}$ and a maximum number of data points $d_{max}$. This way capturing the desired frequency information. Again, features with a saturation at a lower lb and an upper bound ub were implemented. The conditions were defined as

(7) f(xi)=100,  [s=1, w=0,   p=0];ifv>u∧s=0∧w≥dmin∧p<dmax0,  [s=1, w=0,   p=0];elseifv>u∧s=0∧w≥dmin0,  [s=s, w=w+1,   p=p];elseifv>u∧s=00,  [s=1, w=0,   p=p+1];elseifv>u100,  [s=0, w=0,   p=0];elseifv<u∧s=1∧w≥dmin∧p<dmax0,  [s=0, w=0,   p=0];elseifv<u∧s=1∧w≥dmin0,  [s=s, w=w+1,   p=p];elseifv<u∧s=10,  [s=0, w=0,   p=p+1];else,

where *s* denotes the sign of the previous slope. The variables *w* and *p* are counters and *u* and *v* are the smoothed signal from Equation (5):

(8) $$u=smo_{i-1}\;,$$

(9) $$v=smo_{i}\;.$$

#### 2.2.4. Waveform Length (WFL)

Different variants of the feature WFL were implemented. The waveform length is calculated by

(10) $$f\left(x_{i}\right)=\left|x_{i}-x_{i-1}\right|d\;.$$

#### 2.2.5. Mean Absolute Value (MAV)

The function $f(x_{i})$ for the MAV was defined as

(11) $$f\left(x_{i}\right)=\left|x_{i}\right|\;.$$

The MAV2 is calculated by means of the MAV1 by

(12) $$MAV2_{i}=(MAV2_{i-1}+|MAV1_{i}-MAV1_{i-delay}\left|\right)c\;.$$

The feature MAV1S is saturated at the upper bound ub1 and the MAV2S at ub2.

#### 2.2.6. Wilison Amplitude (WAM)

When the EMG signal exceeds a defined threshold thresh, the feature is increased by 100. The conditions for WAM1 were defined as

(13) $$f\left(x_{i}\right)=\left\{\begin{array}{cc} 100; & \mathrm{if}\;|x_{i}|>thresh\\ 0; & \mathrm{else}\;. \end{array}\right.$$

For WAM2, the smoothed signal $smo_{i}$ is calculated with Equation (5) and the conditions are

(14) $$f\left(x_{i}\right)=\left\{\begin{array}{cc} 10; & \mathrm{if}\;|\left(x_{i-delay}d\right)-smo_{i}|>thresh\\ 0; & \mathrm{else}\;. \end{array}\right.$$

#### 2.2.7. Variance (VAR)

The VAR is the squared EMG signal, which was also implemented with a saturation at the upper bound ub. The VAR is calculated by

(15) $$f\left(x_{i}\right)=x_{i}^{2}\;.$$

### 2.3. Downsampling

For the training of the NN, decision tree, and logistic regression, the features were downsampled from 2 kHz to 40 Hz to accelerate model training. In the implementation the features were not downsampled to achieve short time periods between the decisions. For the RNN, the features were downsampled from 2 kHz to 250 Hz, which led to a higher calculation effort in the learning process. However, an equal sampling frequency in the training and implementation is indispensable for high accuracies of RNNs.

### 2.4. Correlation of Features

To reduce the number of features before training, the Pearson correlation coefficients [19] of the feature vectors were calculated by pairwise comparison. For an absolute value of the correlation coefficient greater than 0.9, one feature of the pair was eliminated.

### 2.5. Normalization

For the logistic regression, NN and RNN (but not for the decision tree) the min-max normalization was calculated for each entry of the feature vector feat:

(16) $$feat_{i}^{\prime }=\frac{\left(y_{max}-y_{min}\right)\left(feat_{i}-feat_{min}\right)}{\left(feat_{max}-feat_{min}\right)}+y_{min}$$

with $y_{max}=1$ and $y_{min}=-1$ in floating-point format. The minimum and maximum ($feat_{min}$, $feat_{max}$) were determined for the feature vector calculated with the training data.

### 2.6. Training of Models

All models were trained with a 5-fold cross validation. The validation data were randomly drawn time sequences of the strong, short and weak contractions and the artifacts. The decisions within 100 ms after a transition of the target value were not included in the error calculation of the validation data. Wrong decisions, starting from 100 ms after a transition of the target value, were recorded as errors, thus achieving fast models. For the test data, this tolerance was set to 150 ms (cf. signal delay, Section 2.8.3). The models were trained with functions provided by the MATLAB^®^ toolbox “Statistics and Machine Learning”.

#### 2.6.1. Logistic Regression

The features were selected with the lasso algorithm [20] assuming a binomial distribution. 30 different regularization penalties (λ) were applied. The range of the λ was defined such that the resulting feature vectors were ranging from a maximum number of regression coefficients to maximum sparsity. A vector with the desired number of features was selected and the non-zero entries were the selected features.

With these features the logistic regression model was trained by the Ridge Regression algorithm [21]. Finally, the model with the highest accuracy was selected.

#### 2.6.2. Decision Tree

Binary decision trees were trained with different numbers of maximum splits, which avoids overfitting. The split and the respective feature was selected by calculating the Gini Diversity Index [22].

#### 2.6.3. Neural Network (NN)

The features for the shallow NNs with one hidden layer were chosen by sequential backwards selection [23]. The activation function for the hidden unit was *SATLINS*, and *PURELIN* for the output layer. These activation functions allow a fast calculation on an embedded system. The NNs were trained by the Levenberg-Marquardt algorithm [24,25], aiming at the minimization of the cross-entropy loss [26]. The model was trained with a maximum number of epochs of 1000 and a maximum of 6 validation failures.

#### 2.6.4. Recurrent Neural Network (RNN)

**Figure 6:**
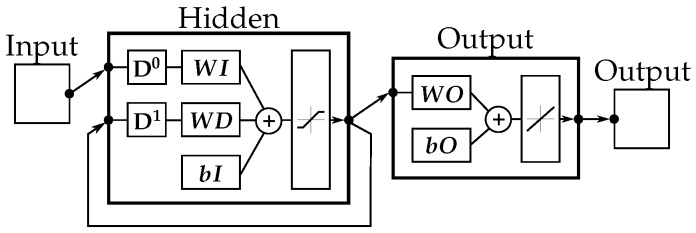
Shallow NN with one recurrent path with a delay of one ($D^{1}$).

For the RNN (Figure 6), the maximum number of epochs was set to 100. All other hyperparameters were set as described in Section 2.6.3.

### 2.7. Offline Data Post-Processing

The model decision was further processed to increase the stability against short wrong decisions. The system should be prevented from incorrect reactions due to individual false classified samples. To allow a transition of the decision, a minimum number of equal consecutive decisions was required. The transition at the output was only conducted after (excl.) $n_{slope}$ equal decisions. For the offline post-processing $n_{slope}$ was set to 2 for the NNs, decision trees and logistic regressions, and to 3 for the RNNs. This parameter introduces an additional delay, which is depending on the model decision frequency and the value of $n_{slope}$. The parameter $n_{slope}$ was selected in a way to achieve similar delays for the different implementations. Despite the additional delay, $n_{slope}$ is essential for a high stability of the system. This parameter value was selected, as it is a good trade-off between delay and stability, however, it can be adapted to the individual amputee’s preference.

### 2.8. Implementation

The features and the models were implemented on an ultra-low-power μC, the ATSAML21E18B from Microchip Technology Inc. (Chandler, AZ, USA) with a 32 bit ARM^®^ CORTEX^®^-M0+ processor, 256 kB flash, and 32 kB SRAM main memory. The clock frequency was set to 48 MHz to test the implementations. The clock frequency can be reduced (depending on the runtime of the selected model) to decrease power consumption [2].

The analog digital sampling of the EMG signal has been described by Roland et al. [2]. The digital signal processing sampling frequency was set to 2 kHz. For the RNN (250 Hz), the 48 MHz clock provides 192,000 clock cycles (4 ms) between two decisions. The NN, decision tree, logistic regression, and the feature calculation (2 kHz) provide 24,000 clock cycles (500 μs) between two samples.

#### 2.8.1. Fixed-Point Representation

All algorithms were implemented in fixed-point format, as described by ARM Ltd. (Cambridge, UK) [27], which is faster than a floating-point implementation on this μC. For high precision and to avoid overflows or saturations the optimal value range of the variables was selected to exploit the full operating range of the data type. The quantization effects and possible overflows or saturations had already been considered in the offline design. The high performance of the μC 32 bit hardware multiplier was exploited with this fixed-point implementation. All divisions were replaced by shift operations to require low calculation resources.

#### 2.8.2. Online Data Post-Processing

As mentioned above, the time between two decisions was deviating in the offline and online implementations for the NN, decision tree and logistic regression. Therefore, different post-processing parameters were selected. The parameter $n_{slope}$ was set to 20 in the implementation of the NN, decision tree and logistic regression. For the RNN, $n_{slope}$ was set to 3, equal to the offline design.

#### 2.8.3. Signal Delay

The filtered EMG signal and the decision were multiplied at the output; therefore, they should correspond to each other in time. Hence, the input signal was delayed, so that it corresponds to the current decision. The smoothing had a time constant *T* of 51 ms [2], which introduced a delay. An additional delay was set to 100 ms in the implementation.

## 3. Results

Table 8 lists the accuracies of the resulting models. Please note that these accuracies were calculated for test data with many transitions. Additionally, many artifacts were in a similar frequency and amplitude range than the contractions. Furthermore, transitions longer than 150 ms were recorded as errors and small signals were removed from the data set. Consequently, these challenging data led to higher error rates in the evaluation but to a highly robust behavior in practical application.

The runtime of the implemented features is shown in Table 9 and of the models in Table 10. The runtime of the decision tree is listed for the shortest and longest branch.

The time constant *T* of the EMA for the features MCR2, WFL2S, WAM1 was 63.5 ms, and 127.5 ms for all other features.

For the logistic regression, NN and RNN, the feature calculation was followed by the normalization to ensure that the features were in an equal value range. For the decision tree, no normalization was required. The coefficients were determined with the features calculated for the training data. These quantized coefficients were implemented on the μC. The resulting runtime for each feature normalization was 0.53 μs.

### Selected Models

**Figure 7:**
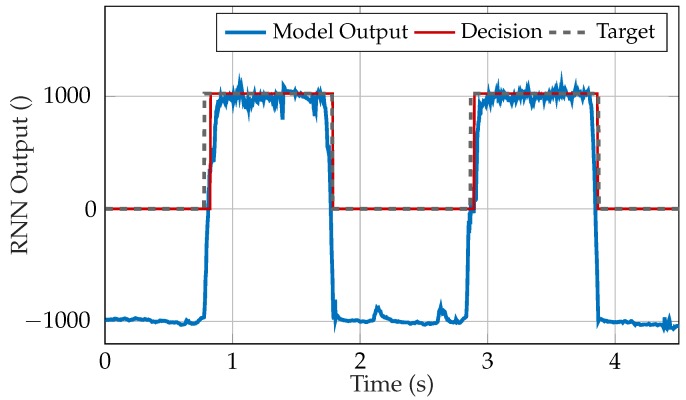
Output, decision and target of the selected RNN for a window of the test data, corrected by the signal delay (Section 2.8.3). The target value is 1024 (= 1 in floating-point format) for contractions and 0 for artifacts. A decision of 1024 leads to an activation of the prosthesis drive. When an artifact is detected, the current output value is maintained or the prosthesis drive is turned off, depending on the selected policy.

The choice of the final model depends on the requirements. For a highly robust system, the author suggests the RNN with 9 hidden units and 9 features. Figure 7 shows the output of the selected model for a window of the test data. The confusion matrix of the test data for this RNN is presented in Table 11.

**Figure 8:**
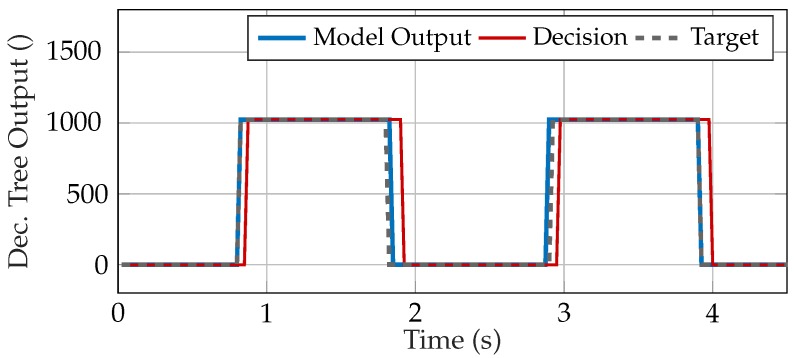
Output, decision and target of the selected decision tree for a window of the test data, corrected by the signal delay (Section 2.8.3). The target value is 1024 (= 1 in floating-point format) for contractions and 0 for artifacts. A decision of 1024 leads to an activation of the prosthesis drive. Decisions for artifacts maintain the current output value or turn off the prosthesis drive, depending on the selected policy.

For an application with minimum calculation effort but high performance, the decision tree with 3 features (SSC3, ZCR2, VAR1S) and 4 splits is the best choice. In comparison to the RNN, the decision tree has a longer time lag at the transitions (see Figure 8), which leads to lower accuracies. By increasing the signal delay, the accuracy could be improved; however, in this article, long transitions were recorded as an error, because it was aimed at a fast system. The confusion matrix of the selected decision tree is presented in Table 12. Please note that the decision tree has less test samples due to a lower sampling frequency, see Section 2.3. However, the durations of the test data time sequences of the decision tree and RNN are equal.

## 4. Conclusions

Prosthesis drive activation due to artifacts is highly unpleasant for amputees. Avoiding incorrect activation leads to higher performance and thereby increases the acceptance of the myoelectric prostheses. With the presented models, the artifacts and contraction EMG signals were successfully distinguished with high accuracies. These algorithms substantially improved the robustness of the flexible insulated EMG sensors in real-world environment. Activation of the prosthesis drive due to artifacts is now effectively prevented by the implemented models. This high performance was achieved also by the algorithms’ fast reaction to signal changes. All the models were designed to require low calculation effort for implementation on low-power wearable systems. With the shallow models, high accuracies were accomplished, although many transitions of the classes were included in the test data. The algorithms led to a short time between decisions as no Fourier transform had to be calculated. The presented models use hand-crafted time domain features only. Due to the wisely designed features, high accuracies were achieved with shallow models.

The author suggests an RNN or a regular NN for highest accuracies (99.91%/99.06%). For an implementation, which requires very little calculation resources, the author suggests a decision tree (98.76% accuracy).

As mentioned above, the linear separation algorithm presented by Roland et al. [16] has difficulties in detecting short contractions. This algorithm is now outperformed by the new models. Additionally, the need for the calculation of the short-time Fourier transform is obviated.

In the next step, models will be trained on amputees and the EMG sensor system with the presented motion artifact suppression will be applied to real-world prosthesis control. Furthermore, the potential of improving the performance by a random forest or a convolutional NN will be evaluated. The accuracies of ensemble models will be investigated. The runtime of the features and the models will be included to the cost term and a global optimization problem, which also comprises the feature parameters, will be solved. Additionally, the application of these algorithms to the state-of-the-art conductive EMG sensors and to other biosignal sensors (e.g., EEG or ECG) will be investigated. Furthermore, a multiple subject dataset will be acquired, and the presented models will be trained with the new data. The effect of inter-subject variability in the EMG signal will be evaluated. It will be examined whether a model trained on multiple subjects or a model trained on the individual EMG data leads to the best performance.

## Figures and Tables

**Table 1:** Parameters of ZCR implementations.

Feature	b	hyst	ub
ZCR1	0.9961	0	2^16^−1
ZCR2	0.9961	242	2^16^−1
ZCR2S	0.9961	242	1000

**Table 2:** Parameters of MCR implementations.

Feature	b	c	d	e	delay	hyst	lb	ub
MCR1	0.9961	0.8750	6	1	8	1044	0	2^16^−1
MCR1S	0.9961	0.8750	6	1	8	1044	2000	3600
MCR2	0.9922	0.9688	1	0.0313	8	0	0	2^16^−1

**Table 3:** Parameters of SSC implementations.

Feature	b	c	$d_{min}$	$d_{max}$	lb	ub
SSC1	0.9961	0.9922	0	2^16^−1	0	2^16^−1
SSC1S	0.9961	0.9922	0	2^16^−1	2000	5000
SSC2	0.9961	0.5	0	2^16^−1	0	2^16^−1
SSC2S	0.9961	0.5	0	2^16^−1	0	14000
SSC3	0.9961	0.9961	5	2^16^−1	0	2^16^−1
SSC3S	0.9961	0.9961	5	2^16^−1	900	2000
SSC4	0.9961	0.9961	0	1	0	2^16^−1
SSC5	0.9961	0.75	3	200	0	2^16^−1
SSC5S	0.9961	0.75	3	200	1500	3000

**Table 4:** Parameters of WFL implementations.

Feature	b	d	ub
WFL1	0.9961	0.0078	2^16^−1
WFL1S	0.9961	0.0078	1300
WFL2S	0.9922	0.0156	1894

**Table 5:** Parameters of MAV implementations.

Feature	b	c	delay	ub1	ub2
MAV1	0.9961	-	0	2^16^−1	2^16^−1
MAV1S	0.9961	-	0	600	2^16^−1
MAV2	0.9375	0.9961	8	2^16^−1	2^16^−1
MAV2S	0.9375	0.9961	8	4000	6000

**Table 6:** Parameters of WAM implementations.

Feature	b	c	d	delay	thresh
WAM1	0.9922	-	-	0	44
WAM2	0.9961	0.9922	16	64	3636

**Table 7:** Parameters of VAR implementations.

Feature	b	ub
VAR	0.9961	2^16^−1
VARS	0.9961	4000

**Table 8:** Accuracies of quantized models in %.

Model\Num. Features	3	6	9	12
NN (3 Hidden Units)	97.24	97.30	97.05	98.95
NN (6 Hidden Units)	97.24	98.95	98.89	98.87
NN (9 Hidden Units)	95.79	97.28	98.68	99.06
RNN (3 Hidden Units)	77.47	96.51	98.66	99.22
RNN (6 Hidden Units)	91.63	97.06	99.16	98.66
RNN (9 Hidden Units)	93.20	98.33	99.91	99.67
Decision Tree	96.96	97.93	98.76	98.18
Log. Regression	94.34	95.22	95.60	95.12

**Table 9:** Runtime of input features in μs at a 48 MHz clock.

ZCR1	**1.97**	ZCR2	**2.05**	ZCR2S	**2.05**	MCR1	**3.62**
MCR1S	**3.62**	MCR2	**3.55**	SSC1	**2.42**	SSC1S	**2.42**
SSC2	**2.42**	SSC2S	**2.42**	SSC3	**2.42**	SSC3S	**2.42**
SSC4	**2.42**	SSC5	**2.42**	SSC5S	**2.42**	WFL1	**2.57**
WFL1S	**2.57**	WFL2S	**2.57**	MAV1	**2.05**	MAV1S	**2.05**
MAV2	**5.42**	MAV2S	**5.42**	WAM1	**2.95**	WAM2	**3.77**
VAR	**2.27**	VARS	**2.12**			

**Table 10:** Runtime of models in μs at a 48 MHz clock.

Model\Num. Features	3	6	9	12
NN (3 HU)	12.16	15.16	17.78	20.77
NN (6 HU)	18.53	24.14	29.39	35.28
NN (9 HU)	24.89	33.13	41.75	49.99
RNN (3 HU)	19.65	23.02	26.39	29.76
RNN (6 HU)	38.75	45.49	52.23	58.98
RNN (9 HU)	64.22	74.56	84.82	94.93
Decision Tree	0.99–1.15	0.99–1.30	1.15–1.60	1.15–1.67
Log. Regression	1.67	2.12	2.87	3.62

**Table 11:** Confusion matrix for selected RNN model (9 hidden units, 9 input features).

$N_{TestSamples}=$16,008	Predicted	Predicted
	Contraction	Artifact
Actual Contraction	7665	3
Actual Artifact	12	8328

**Table 12:** Confusion matrix for selected decision tree (3 input features).

$N_{TestSamples}=$4774	Predicted	Predicted
	Contraction	Artifact
Actual Contraction	2215	65
Actual Artifact	80	2414
